# Occurrence of *pfatpase6* Single Nucleotide Polymorphisms Associated with Artemisinin Resistance among Field Isolates of *Plasmodium falciparum* in North-Eastern Tanzania

**DOI:** 10.1155/2015/279028

**Published:** 2015-01-05

**Authors:** Jaffu Chilongola, Arnold Ndaro, Hipolite Tarimo, Tamara Shedrack, Sakurani Barthazary, Robert Kaaya, Alutu Masokoto, Debora Kajeguka, Reginald A. Kavishe, John Lusingu

**Affiliations:** ^1^Kilimanjaro Christian Medical University College and Kilimanjaro Clinical Research Institute, Moshi, Tanzania; ^2^Sokoine University of Agriculture, Morogoro, Tanzania; ^3^National Institute for Medical Research, Tanga, Tanzania

## Abstract

We aimed to determine the current prevalence of four *P. falciparum* candidate artemisinin resistance biomarkers L263E, E431K, A623E, and S769N in the *pfatpase6* gene in a high transmission area in Tanzania in a retrospective cross sectional study using 154 archived samples collected from three previous malaria studies in 2010, 2011 and 2013. Mutations in *pfatpase6* gene were detected in parasite DNA isolated from Dried Blood Spots by using PCR-RFLP. We observed overall allelic frequencies for L263E, E431K, A623E, and S769N to be 5.8% (9/154), 16.2% (25/154), 0.0% (0/154), and 3.9% (6/154). The L263E mutation was not detected in 2010 but occurred at 3.9% and 2.6% in 2011 and 2013 respectively. The L263E mutation showed a significant change of frequency between 2010 and 2011, but not between 2011 and 2013 (*P* < 0.05). Frequency of E431K was highest of all without any clear trend whereas S769N increased from 2.2% in 2010 to 3.6% in 2011 and 5.1% in 2013. A623E mutation was not detected. The worrisome detection and the increase in the frequency of S769N and other mutations calls for urgent assessment of temporal changes of known artemisinin biomarkers in association with *in vivo* ACT efficacy.

## 1. Introduction

Resistance to antimalarial drugs is a major public health problem, which hinders the control of malaria. Widespread* P. falciparum* resistance to conventional antimalarial medicines such as chloroquine and sulphadoxine-pyrimethamine (SP) led to a change in malaria treatment policy in endemic countries [1]. ACTs have become the mainstay of falciparum malaria therapy in endemic areas [2]. Despite their excellent curative rate, reports of possible* P. falciparum* resistance to ACTs in Senegal, in French Guiana, and on the Thai-Cambodia border have recently been reported [3, 4] which suggests a probable decline in the effectiveness of ACTs and artesunate monotherapies against malaria. Although ACTs have clearly proven effective for the treatment of malaria in most endemic areas so far, concerns remain about their long-term implementation as first-line therapy [2]. This concern is exacerbated by the absolute dependence on artemisinin derivatives in virtually all of the existing antimalarial combination therapies, creating a substantial selection advantage for parasites with decreased susceptibility to artemisinins. SNPs in the Pfmdr-1, Pfcrt, and Pfdhfr genes have also been suggested to be markers of resistance to various antimalarial drugs, including ACTs [5, 6]. However, it has previously been suggested that a sarcoplasmic-endoplasmic reticulum Ca^2+^-ATPase- (SERCA-) type protein, encoded by the* pfatpase6* gene, is an important chemotherapeutic target of artemisinins [7–11]. Moreover, previous studies have proposed that single nucleotide polymorphisms (SNPs) in the* pfatpase6* gene affect* in vitro* sensitivity to artemisinins [8–10]. In addition, previous studies have shown a considerable increase in artemether IC_50_ with* pfatpase6* SNPs (L263E, E431K, A623E, and S769N), particularly, the* pfatpase6* S769N mutation [12]. The L263E mutation and the combination of two* pfatpase6* SNPs, E431K and A623E, have been shown to affect inhibition of PfSERCA by artemisinin [13, 14]. Many other SNPs have been identified as possible markers for artemisinin resistance although their validity is still under investigations. At a time when worrisome reports of reduced parasite sensitivity to ACTs are published from South East Asia and elsewhere [4, 15, 16], limited data is available on the status of resistance to ACTs in the Eastern Africa region since ACTs adoption about 10 years ago. The potential of molecular markers for predicting therapeutic efficacy has already been documented and demonstrated [5, 6, 12, 15, 17, 18]. In this study, we aimed to determine the prevalence of SNPs in the* pfatpase6* gene at codons 263, 431, 623, and 769 in four high malaria transmission villages in Bondo ward in Handeni in North Eastern Tanzania, for changes in their frequencies over time between 2010 and 2013.

## 2. Methodology

### 2.1. Study Area, Procedures, and Sample Processing

The study was retrospective cross-sectional study whereby samples collected from three different cross-sectional malaria studies in 2010, 2011, and 2013 in Bondo Village were used. Bondo village is located in Handeni District in the coastal region of Tanga, North-Eastern Tanzania. The region is endemic to malaria with a perennial transmission. It is known to be a focal area for malaria drug resistance due to the drug selection pressure [19]. The study area has two rainy seasons per year which denotes the peaks of malaria transmission. The prevalence of malaria was 23% in 2013 (unpublished data). Dried blood spots (DBS) collected from microscopically confirmed malaria positive individuals were used. Only DBS with proper labels of date of collection and malaria positivity were included. A total of 154 DBS were selected and used in the study, 50, 52, and 52 from the 3 surveys in 2010, 2011, and 2013, respectively.

### 2.2. Isolation of* P. falciparum* Genomic DNA and PCR-RFLP

DNA was extracted from DBS using chelex100-saponin method as previously described earlier [20]. PCR reactions were carried out using a T100 Thermo Cycler (Biorad Laboratories Inc, San Francisco, CA, USA). Malaria parasite DNA was extracted using chelex-100 method as described previously [21]. Genotyping for ATPase6 SNPs was performed using PCR-RFLP methods as described elsewhere [20]. All PCR reagents and restriction endonucleases were purchased from Carramore (Holmfirth, Thongsbridge Mills, UK). Amplification of the* pfatpase6* gene was carried out in a final volume of 25 *μ*L containing 2 *μ*L of extracted DNA, 250 nM primers, Taq buffer, 2 mM MgCl_2_, 125 *μ*M each of the four deoxyribonucleotide triphosphates (dNTPs), and 0.4 U High Fidelity Platinum Taq polymerase. Secondary PCR reactions were carried out in similar conditions as the primary PCR except that 1 uL of primary PCR product was used as template. Conditions for restriction digestion, amplicon sizes and enzymes, are shown in Table 1. Secondary PCR products were digested with four enzymes each for a specific SNP:* Apo*I,* MBo*II,* Cac8*I, and* Dde*I for detection of mutations at positions L263E, E431K, A623E, and S769N, respectively. Restriction digestion was carried out as described by manufacturer. Restriction products were resolved on 1.5–3% agarose gel containing ethidium bromide and visualized under UV florescent illuminator, as a way of quality assurance.

### 2.3. Ethics

Ethical approval for this study was granted by the Kilimanjaro Christian Medical University College Ethics and Research Committee (CRERC) with a certificate number 554.

## 3. Results

*Pfatpase6* gene was successfully amplified and genotyped in all 154* P.  falciparum* field isolates. Detection of* pfatpase6* SNPs within positions L263E, E431K, A623E, and S769N indicated 5.8% (9/154), 16.2% (25/154), 0.0% (0/154), and 3.9% (6/154) overall prevalence of* pfatpase6* mutant genotypes (both heterozygous and homozygous mutant combined), respectively. The homozygous allelic frequency was distributed such that, for L263E, E431K, A623E, and S769N, frequencies were 8 (1.4%), 17 (11.0%), 0 (0.0%), and 5 (3.2%), respectively. Majority of parasites had the wild type allele: L263E, 145 (94.2%); E431K, 125 (81.2%); A623E, 0 (100%); and S769N, 148 (96.4%).

We analyse data to determine allelic frequency changes in the period from 2010 to 2013. Results for analyses that combined heterozygous and homozygous allelic distribution are presented in Table 2 and Figure 4. From this analysis, the L263E mutation was not detected in parasite isolates collected in 2010; however, it occurred at frequency of 6 (3.9%) and 4 (2.6%) in 2011 and 2013, respectively. The E431K mutation occurred at 26 (16.9%), 16 (10.4%), and 20 (13.0%) in 2010, 2011, and 2013, respectively. The A623E mutation was not detected in any of the isolates analyzed. We observed that the frequency of the S769N mutation was 3 (1.9%) in 2010, 6 (3.9%) in 2011, and 8 (5.2%) in 2013. Generally, the E431K mutation was the most frequent allele by 16.2%, followed by L263E (5.8%), S769N (3.9%), and A623E which was not detected. Occurrence of more than one of the studied mutations was rare, in which case only E431K and S769N occurred in 2 isolates in the years 2011 and 2013.

We explored data for all four SNPs to determine if there was a significant change in frequency between the years 2010 and 2013 using Fisher's test. To accomplish this analysis, we combined heterozygous and homozygous mutants for the analyses and data are presented in Table 3. We could not demonstrate a significant change in prevalence of any of the SNPs between all three years 2010 through 2013. This is evidenced by the overlapping 95% CI values across the years shown in Table 3. However, the L263E mutation showed the highest change across the three years, which was closest to significance (*χ* ^2^ = 5.7239, *P* = 0.05716). Pairwise comparisons of frequencies by Fisher's exact test revealed a significant change of L263E frequency only between 2010 and 2011, but not between 2011 and 2013 (*P* < 0.05).

## 4. Discussion

Monitoring of potential biomarkers for antimalarial drug resistance is a sustainable and relatively cheap tool for monitoring emergency of drug resistance early enough before* in vivo* efficacy studies are conducted. Many previous studies have reported molecular markers for ACT partner drugs resistance (lumefantrine and amodiaquine and even quinine [20, 22–27]). Artemisinin is thought to inhibit* pfatpase6* of the parasite [7, 9, 14, 28].

**Figure 1:**
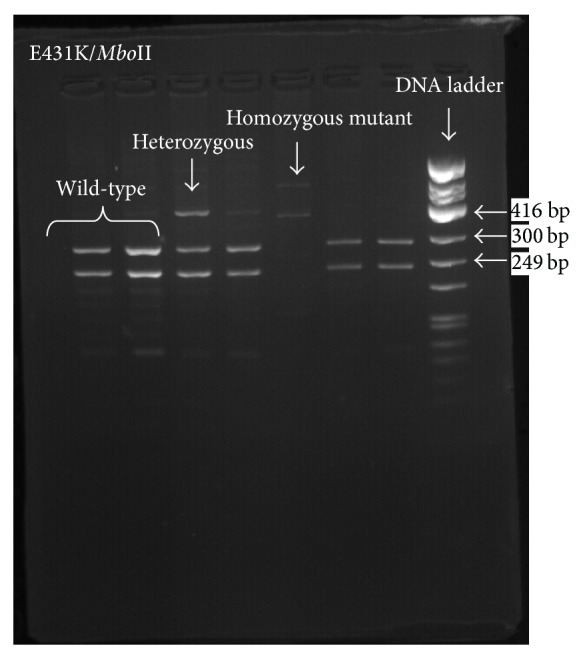
Gel electrophoresis for* pfatpase6* E431K mutation. Agarose gel electrophoresis showing PCR-RFLP product digested with* Mbo*II enzyme for E431K mutation detection showing wild type, homozygous mutant, and heterozygous genotypes.

**Figure 2:**
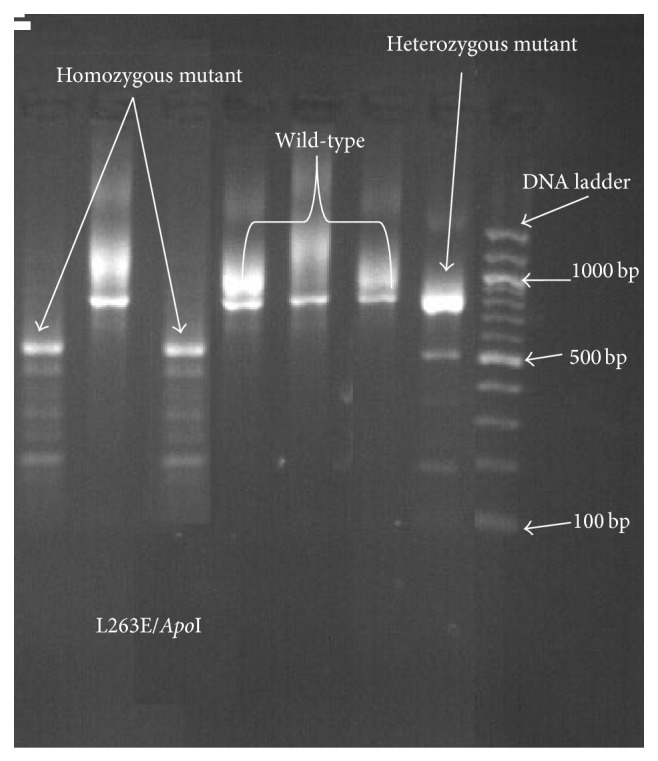
Gel electrophoresis for* pfatpase6* L263E mutation. Agarose gel (2%) electrophoresis showing PCR-RFLP products digested with* Apo*I enzyme to detect the L263E mutation showing the wild type, homozygous mutant, and heterozygous genotypes.

**Figure 3:**
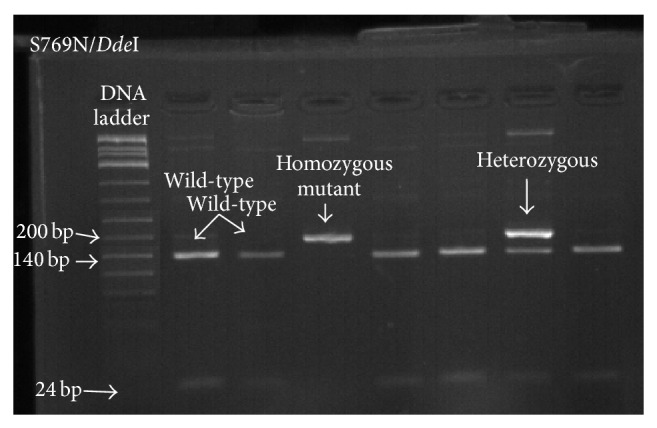
Gel electrophoresis for* pfatpase6 S769N* mutation. Agarose gel electrophoresis showing PCR-RFLP products digested with* Dde*I enzyme to detect the S769N mutation showing the wild type, homozygous mutant, and heterozygous genotypes.

**Figure 4:**
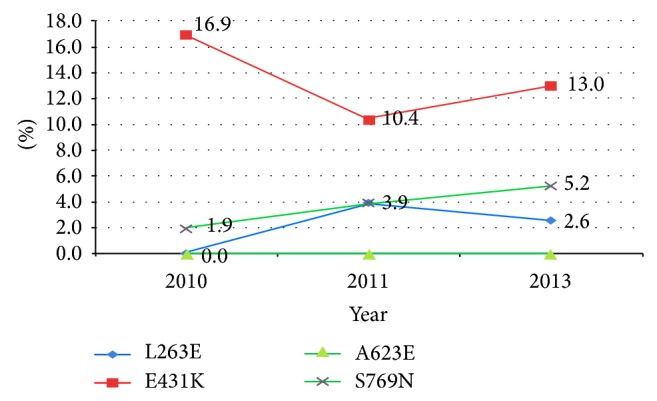
Allelic frequencies of* pfatpase6 mutant* alleles by year, combining homozygous and heterozygous genotypes. All percentages are presented as* n*/154 and homozygous mutants and heterozygous mutants are combined as mutants.

We detected L263E, E431K, and S769N mutations at varying frequencies over the period from 2010 to 2013, a finding that supports previous reports on the high diversity of the* pfatpase6* gene (Figures 1, 2, and 3). Our findings also support previous arguments that many point mutations in the* pfatpase6* gene may exist in one geographical location [3, 9]. Previous studies in Tanzania and Brazil and China have not detected the S769N mutation in field isolates [8, 9, 11]. We see in the current study that the frequency of the mutation shows an increasing pattern from 2010 to 2013. To the best of our understanding, this is the first time this mutation is detected in Tanzania and the Eastern African region. The detection and observed increase in the prevalence of the S769N mutation in the study region are both interesting and worrisome since it may imply early signs of selection of resistant parasites and possible emergency of artemisinin resistance. We did not detect any isolate with the A623E mutation in this study, consistent with previous studies previously done in Tanzania [11].

The L263E mutation was not detected in samples collected in 2010 but emerged in 2011 at a frequency of 3.9%. Previous studies reported this mutation to have been studied mainly in DNA manipulation experiments and not* in vivo* [29, 30]. The detection for the first time of the L263E mutation in the current study using field parasite samples may have many implications including intense drug selection pressure of mutant strains in the study area. The role of the L263E mutation in conferring ACT resistance/reduced responses by field* P. falciparum* isolates needs to be monitored and further validated.

The E431K mutation occurred at the highest frequency compared to other studied mutations. This is similar to what was reported in previous studies done in Cameroon and Iran [21, 31, 32]. The high frequency of the E431K mutation and the absence of a consistent trend in its frequency from year 2010 through 2013 indicate its common occurrence in the study area with a doubtful previously proposed association with drug resistance. The mutation was previously reported, in* in vitro* studies, to be associated with increased artesunate IC_50_ in Senegal and elsewhere [9, 14] although a study in Iran showed a high frequency of the E431K mutation in both ACT exposed and ACT unexposed parasites, indicating its questionable role in ACT resistance [21]. The E431K mutation is the most common mutation of the* pfatpase6* gene found in many African and Asian countries including Tanzania, but its direct association with ACTs resistance has been reported to depend on its co-occurrence with other SNPs in the pfatpase6 gene, usually the L623E mutation (NB change A623E to L623E) [32].

Our data show a sudden increase of L263E and S769N SNPs from 2010 to 2013. The relatively fast increase of the two SNPs within a short period of time from 2010 to 2013 may have a number of different explanations. For many years, the study area had experienced perennial and holoendemic malaria transmission. However, in recent years, malaria transmission has declined substantially and entomological inoculation rate has declined from 148 infectious bites per person per year in 2000 [33] to 39 infectious bites per person per year in 2013 (unpublished data, 2013). The traditional debate on the stimuli responsible for emergency of resistant strains of malaria parasites proposes either host immunity or the drug pressure [16] or both. Other studies have linked transmission intensity and drug resistance [34, 35] with fitness competitiveness between wild and mutant strains [16]. Previous work has also provided evidence that antimalarial resistance emerged first in low transmission areas and spread to other parts [33, 34].

Based on the low transmission theory, the rapidly declining transmission patterns in the study area and neighboring areas may partly explain the rapid increase in the frequencies of two of the studied SNPs which are more closely associated with antimalaria resistance. The renewed and sustained reinforcement of malaria control programs in Tanzania based on both ACT chemotherapy and integrated vector control may have contributed greatly to the decline in malaria transmission and hence favor the increase of resistant strain frequencies. This argument is supported by findings from a recent study conducted in Kenya that reported a significant decline of early response rates of* P. falciparum* infections to ACTs three years after they were introduced [36].

Many factors could commonly contribute to the key mechanisms of emergence of resistant parasite strains in the study area, in common with other relatively underdeveloped malaria endemic regions. Most important is the poor reinforcement of the concept of rational therapy. Improper use of antimalaria drugs is based on presumptive, clinical diagnosis alone or on misdiagnosis due to poor technical expertise on interpretation of laboratory results [2, 37, 38]. Due to incompetence of health delivery systems in many malaria endemic countries, health ministries have made artemisinins available to the private sector to increase patients' access to the drug [39]. Good as it is, this approach has, however, increased the risks that drug use will be uncontrolled [40, 41] since adherence and indication are not properly controlled.

The prevalence of counterfeit or clinically substandard ACTs that contain small quantities of the artemisinin derivatives threatens to subvert ACT efficacy as it provides an ideal mechanism for the selection of resistance. Recent estimates show that 33%–53% of all ACT tablets in mainland Southeast Asia region are counterfeit [42]. A previous study carried out in six African countries also documented substandard medicines in 35% of ACTs purchased from private pharmacies and found artemisinin monotherapy to be common despite the appeal by WHO to halt its production [39]. Lastly, while almost all previously reported antimalarial resistance started in Southwest Asia and spread to other parts of the world, antimalarial resistance in Tanzania has almost always started in the study area and spread to other parts of the country. This augments the need to urgently evaluate ACT efficacy status in the North Eastern region of Tanzania.

## 5. Conclusion

Although our observations are based on molecular detection of resistance biomarkers only, more studies that associate biomarkers with ACT clinical efficacy should be conducted to ascertain the current status of ACT effectiveness. Regular monitoring of ACT resistance biomarkers should continue in order to detect the earliest signs of possible emergency of ACT resistance.

## Figures and Tables

**Table 1:** RFLP conditions used for digestion.

Size (size)	Position	Restriction enzyme	Product size (bp)	
775	L263E	*Apo*I	L: 775	E: 633 + 142
	E431K	*MBo*II	E: 299 + 241 + 117 + 69 + 49	K: 416 + 241 + 69 + 49
141	A623E	*Cac8*I	A: 114 + 27	E: 141
164	S769N	*DdeI *	S: 136 + 28	N: 164

**Table 2:** Trends of *pfatpase6 mutation* frequencies from 2010 to 2013 expressed as *n*/154 (%).

SNP	*Pfatpase6* genotype	2010	2011	2013	Overall frequency
L263E	Wild type	154 (100)	148 (96.1)	150 (97.4)	145 (94.2)
	Heterozygous	0 (0)	0 (0)	0 (0)	2 (1.3)
	Mutant	0 (0)	6 (3.9)	4 (2.6)	8 (5.2)
	H ∗ M	0 (0)	6 (3.9)	4 (2.6)	9 (5.8)

E431K	Wild type	128 (83.1)	138 (89.6)	134 (87.0)	125 (81.2)
	Heterozygous	9 (5.8)	6 (3.9)	4 (2.6)	8 (5.2)
	Mutant	18 (11.7)	11 (7.1)	16 (10.4)	17 (11.0)
	H ∗ M	26 (16.9)	16 (10.4)	20 (13.0)	25 (16.2)

A623E	Wild type	154 (100)	154 (100)	154 (100)	154 (100)
	Heterozygous	0 (0)	0 (0)	0 (0)	0 (0)
	Mutant	0 (0)	0 (0)	0 (0)	0 (0)
	H ∗ M	0 (0)	0 (0)	0 (0)	0 (0)

S769N	Wild type	150 (97.4)	148 (96.1)	146 (94.8)	148 (96.1)
	Heterozygous	2 (1.3)	0 (0)	0 (0)	1
	Mutant	2 (1.3)	6 (3.9)	8 (5.2)	5 (3.2)
	H ∗ M	3 (1.9)	6 (3.9)	8 (5.2)	6 (3.9)

H ∗ M: mutant and heterozygous genotype frequencies combined.

**Table 3:** Analyses for statistical change of pfatpase6 SNPs frequencies from 2010 to 2013.

SNP	Measure	2010	2011	2013
^*δ*^L263E	%	0.0	3.9	2.6
	*N* = *n*/154	0^*^	6^*^	4
	95% CI	0.0–3.0	1.6–8.7	0.8–6.9

E431K	%	16.9	10.4	13
	*N* = *n*/154	26	16	20
	95% CI	11.5–23.9	6.2–16.6	8.3–19.6

A623E	%	0.0	0.0	0.0
	*N* = *n*/154	0	0	0
	95% CI	0.0–3.0	0.0–3.0	0.0–3.0

^*δ*^S769N	%	2.0	3.9	5.2
	*N* = *n*/155	3	6	8
	95% CI	0.5–6.1	1.6–8.7	2.4–10.3

^*^Fisher's exact test revealed a significant change between the two points (*P* < 0.05).

^*δ*^Combined, the frequency change of L263E and S769N over the three years was statistically significant by Fisher's exact test (*P* < 0.05).
